# Regulation of CDK4

**DOI:** 10.1186/1747-1028-1-25

**Published:** 2006-11-08

**Authors:** Laurence Bockstaele, Katia Coulonval, Hugues Kooken, Sabine Paternot, Pierre P Roger

**Affiliations:** 1 Institute of Interdisciplinary Research (IRIBHM), Faculté de Médecine, Université Libre de Bruxelles, Campus Erasme, B-1070 Brussels, Belgium

## Abstract

Cyclin-dependent kinase (CDK)4 is a master integrator that couples mitogenic and antimitogenic extracellular signals with the cell cycle. It is also crucial for many oncogenic transformation processes. In this overview, we address various molecular features of CDK4 activation that are critical but remain poorly known or debated, including the regulation of its association with D-type cyclins, its subcellular location, its activating Thr172-phosphorylation and the roles of Cip/Kip CDK "inhibitors" in these processes. We have recently identified the T-loop phosphorylation of CDK4, but not of CDK6, as a determining target for cell cycle control by extracellular factors, indicating that CDK4-activating kinase(s) might have to be reconsidered.

## Background

At least during embryonic development in mice, CDK4 and its cousin CDK6, as well as their common regulatory partners the three D-type cyclins, are not absolutely required for cell cycle progression [1,2]. E-type cyclins and CDK2 are similarly dispensable for embryo cell proliferation [3-6]. However, loss of CDK4 and CDK2 together (but not combined loss of CDK6 and CDK2 [1]) results in suppressed pRb phosphorylation at midgestation, more severe size reduction in the different organs, embryonic lethality due to heart failure and severe impairment of embryo cell proliferation in culture [7]. Previously, silencing of CDK2 was found to block proliferation in triple cyclin D knockout embryo fibroblasts but not in their wild-type counterparts [2]. This demonstrates the limits of the once unexpected plasticity of cell cycle regulation in mammalian embryo development and of the possible compensation of functions operated by both kinds of G1 phase cyclin-CDK complexes.

That does not mean that cell cycle progression in adult differentiated tissues could normally occur without CDK4 and/or CDK6. For instance CDK4 is dispensable for prenatal development of the pituitary but indispensable for postnatal proliferation of somato/lactotrophs [8]. Similarly, cyclins D2 and D1 are essential for postnatal but not prenatal pancreatic beta-cell growth [9]. Another good illustration is the appearance of the cell cycle requirement for cyclin D3 associated with the maturation of T lymphocyte [10] or late stages of neutrophil development [11]. Indeed, first cell divisions in the mammalian embryo depend on maternal transcripts and proteins, and then in most differentiating lineages cell cycle might proceed without most of the restrictions that control adult cell proliferation [12]. Many embryo cell cycles have a short G1 phase and do not pause in a G0 stage, and thus all the cell circuitry involved in the signalling of cell cycle re-entry from G0 might be partly dispensable until relatively late developmental stages. CDK1 can be activated by cyclin E and thus can compensate CDK2 loss in mice development [13], leading to the perhaps oversimplified vision of the embryonic cell cycle as a basic module resembling the yeast cell cycle that depends on only one CDK. During differentiation, different combinations of the six possible cyclin D1/2/3-CDK4/6 complexes may become adjunct to (plugged in) this core cell cycle module to couple its execution to the various intracellular cascades that transduce signals needed in the whole organism homeostasis.

During later stages of development and in adults, D-type cyclins CDK4/6 complexes thus appear to play essential, yet facilitative, roles as master integrators of the various mitogenic and antimitogenic signals conveyed by the extracellular environment. They are also regulated in response to intracellular mechanisms that sense the metabolic state of the cell, including its energetic and biosynthetic status, and respond to various stresses. According to their tissue-specific, but greatly overlapping, expression patterns, individual D-type cyclins have been shown to be required for proliferation and development of specific tissues (as reviewed in [14]), such as mammary gland and retina for cyclin D1, gonads, B-lymphocyte and pancreatic β-cells for cyclin D2, and maturation of T-lymphocytes for cyclin D3. Like cyclin D1-null mice, CDK4-null mice are smaller, but additionally display abnormalities seen in cyclin D2-deficient mice, including impaired development of pancreatic β-cells [15], whereas CDK6-null mice suffer from haematopoietic defects (reviewed in [14]). It remains somewhat unclear if these specific phenotypes depend only on the particular tissue-specific expression profiles of the various D-type cyclins and their association with CDK4 or CDK6, or might also be related to some non-overlapping functions of their different complexes. At least partial compensation between different D-type cyclins has been well demonstrated [16-18], but it has not been systematically explored in adult tissues. It has recently appeared that CDK6 cannot compensate CDK4 loss in a CDK2-null background [19], but CDK6 might have specific functions in blocking some differentiation processes [20]. Differentiation is often associated with dramatic D-type cyclins' expression switches. In several adult mammalian tissues, cyclin D1 localizes to proliferative layers, whereas cyclin D3 is often highly expressed in the adjacent compartment where differentiation takes place [21-24]. Cyclin D3 replaces cyclin D1 during myogenesis [25,26], adipogenesis [27], or at the pre-TCR developmental stage during T lymphocyte maturation [10].

The six D-type cyclin-CDK4/6 complexes phosphorylate and inactivate the cell cycle/tumor suppressor proteins of the pRb family (p105^*Rb*^, p107, p130^*Rb*2^) [28-33]. This function is indeed essential since the CDK4/6 inhibitor p16 or neutralization of cyclin D1 do not prevent cell cycle progression in pRb-defective cells [34-36]. However, there are also some indications that CDK4/6 can phosphorylate other substrates. Recently, CDK4 (and CDK2) was demonstrated to phosphorylate and inactivate Smad3 [37]. Direct phosphorylations by CDK4 of the nucleolar transcription factor upstream binding factor (UBF) [38], the replication licensing factor Cdt1 [39], the bone-specific transcription factor Runx2 [40], and possibly TSC2 [41] have been reported. Differential phosphorylation of unidentified substrates by cyclin D1-CDK4 or cyclin D3-CDK4/6 was observed [42]. Moreover, cyclin D3 complexes more efficiently phosphorylate p130 than do cyclin D1 complexes [43]. Finally, CDK4 and CDK6 may have different preferences for phosphorylation of specific sites in pRb protein [44], and we have recently found distinct specificities of pRb phosphorylation by CDK4 activated by cyclin D1 or cyclin D3 [45].

CDK4 activity is deregulated in many human tumors [46,47]. It has been recently confirmed to be crucial for various oncogenic transformation processes [2,48-53]. Deregulation does not only result from oncogenic hyperactivation of mitogenic signalling cascades ending at D-type cyclin gene transactivation. Every particular component of "the Rb pathway" act as tumor suppressors or protooncogenes (reviewed in [14,46,47]). Amplification or rearrangement of the genes encoding cyclins D1, D2 and D3 have been found in many human cancers and leukaemias. Oncogenic mutations of cyclin D1 enforcing its nuclear accumulation have been recently described [54]. Loss of p16 by mutation, deletion or gene silencing is extremely frequent. CDK4 itself is overexpressed as a result of gene amplification, or subject to a mutation that renders it insensitive to p16. Interesting models of CDK4/6 deregulation are also provided by some oncogenic viruses. Cyclin K encoded by Kaposi's sarcoma-associated herpesvirus strongly activates CDK6 and CDK4 through original mechanisms [55], and the Tax oncoprotein of human T-cell leukaemia virus type 1 directly binds to and activate CDK4, which involves enhanced cyclin D interaction and suppression of inhibition by p16 [56,57].

## CDK4 regulation

The so widespread deregulation of CDK4 in cancers underscores the necessity to fully understand the various mechanisms involved in its activation process. As initially considered, mitogens activate CDK4/6 by inducing at least one D-type cyclin (D1, D2 and D3) to concentrations allowing to overcome an inhibitory threshold imposed by INK4 CDK4/6 inhibitory proteins [58]. These proteins (p15, p16, p18, p19) bind to the catalytic domain of the isolated CDK4/6, thus preventing cyclin association and activation [59-61]. In short, the synthesis of cyclin D1 and cyclin D2 is stimulated by various mitogenic factors and repressed by growth inhibitory treatments (though cyclin D3 is often associated with quiescent cells [21]). On the opposite, the accumulation of INK4 inhibitory proteins is increased in anti-proliferative situations, such as senescence (p16), TGFβ treatment (p15)[62] or specific differentiation processes (p18 and p19)[63]. In some systems, CDK4 expression itself is increased, which can potentially titrate INK4 proteins, explaining for instance that overexpression of CDK4 can relieve TGFβ inhibition of proliferation [64].

Increased expression of a D-type cyclin is clearly not sufficient to activate CDK4. In this overview, we will address various molecular features of CDK4 activation that are critical but remain poorly known or debated, including the regulation of its association with D-type cyclins, its subcellular location, its activating Thr172-phosphorylation and the roles of Cip/Kip CDK "inhibitors" in these processes. We will often evocate questions raised by the S-phase entry in a specific model system that we have characterized, the primary culture of canine thyroid epithelial cells [65,66]. Indeed, this physiologically relevant model displays several interesting features, including the coexistence of two very distinct mitogenic pathways [67,68], which both require CDK4 activity [69,70] : (i) a canonical mitogenic stimulation by growth factors associated with reversible dedifferentiation, and (ii) a mitogenic stimulation associated with enhanced thyroid function and differentiation expression by the physiological stimulus, TSH, which signals only through elevation of cellular cyclic AMP levels without activating most of the classical intermediates of growth factor-dependent cascades (including Ras, ERKs and PI3-kinase pathways)[65,71-73]. Paradoxically, this cAMP-dependent triggering of cell cycle progression involves an accumulation of the p27^kip1 ^CDK inhibitor [74] but not of the D-type cyclins [70], as observed in cell systems where cAMP blocks G1 phase progression [75,76]. Cyclin D3 is nevertheless required in this differentiation-compatible mitogenic stimulation, but not for the proliferation stimulation by growth factors that induce the other D-type cyclins [70]. This specific requirement for cyclin D3 is consistent with the emerging concept that cyclin D3 might be more specifically involved in specialized cell cycles associated with maintenance or induction of differentiation [10,21,45].

### Regulated assembly of D-type cyclin-CDK4 complexes

Early studies by the group of Sherr have shown that D-type cyclins and CDK4 do not assemble spontaneously in vitro, but nevertheless can form active complexes when marinated with an extract of proliferating NIH3T3 cells, but not (as reported but not shown) with extracts of growth-factor deprived cells [77]. Since the association of CDK4 with the cyclin D was not shown in these experiments, this assay could not discriminate between assembly and activation activities. Nevertheless, using NIH 3T3 cells engineered to constitutively express both cyclin D3 or cyclin D1 and CDK4, the same authors showed that the formation of cyclin D1/3-CDK4 complexes depends on serum stimulation [78], which can be replaced by expression of an activated form of MEK1 [79]. However, in a similar experimental setting, Ladha et al [80] found that overexpressed cyclin D1 can efficiently assemble with endogenous CDK4 to form inactive complexes in serum-deprived NIH 3T3 cells. Cyclin D3-CDK4 complexes are also constitutively assembled in G_0 _cells such as serum-deprived Balb c 3T3 cells [81] and T98G cells [82].

The dog thyrocyte system has provided the first evidence for a critical regulation of D-type cyclin-CDK4 assembly by a physiological mitogenic stimulus [70]. In these cells, cyclin D3 is abundantly expressed depending in part on the presence of comitogenic factors (insulin and carbachol), whereas CDK4 is constitutively expressed. TSH and cAMP trigger the entry into S-phase at least in part by promoting the assembly of required cyclin D3-CDK4 complexes [70,83-85]. Other examples of regulation of the formation of D-type cyclin-CDK4/6 complexes unexplained by modulations of cyclin D or CDK4/6 presence were found in B lymphocytes [86], or FSH-stimulated granulosa cells of hamster preantral follicles [87].

Such findings led to the suggestion that an assembly factor might be required for the assembly of cyclin D-CDK4 complexes [77,78]. In recent years, several interactors of cyclins D or CDK4/6 have been proposed to play a role in the formation of their complex. They are discussed below:

#### p21/p27

At variance with initial reports (reviewed in [59]), Cip/Kip CDK inhibitors (p21^*cip1*^, p27^*kip1*^) have been paradoxically found to be associated with a pRb-kinase activity ascribed to CDK4 [82,88-91]. As they possess distinct binding domains for cyclins and CDKs, they were proposed as the elusive adaptors for the assembly of D-type cyclin-CDK complexes. Indeed, LaBaer and colleagues found that p21 and p27 can promote the formation of these complexes in vitro or in cotransfected cells (yet only p21 could support the pRb-kinase activity) [90]. p21 and p27 were even concluded to be essential for this function, on the basis of the lack of detectable D-type cyclin-CDK complexes in p21/p27-null mouse embryonic fibroblasts [92]. Nevertheless, these authors noticed that phosphorylation of pRb on Ser780 (a site specifically phosphorylated by CDK4/6) was unaffected. Moreover, D-type cyclin levels were also much reduced, which may in part explain the failure to detect their complexes with CDK4/6 [92]. Indeed, in similar experimental settings other authors more recently reported that p21 and p27 do stabilize cyclin D3-CDK4 [93] and cyclin D1-CDK4 [94], but are not required for the assembly of these active complexes, which are inhibited by p16. Moreover, Pledger's group claimed that only the minor fraction of cyclin D3-CDK4 complexes devoid of CIP/KIP proteins are active as pRb-kinases [95].

The case for p21 as a positive regulator of D-type cyclin-CDK4 complexes is nevertheless well supported. In various cell systems, p21 is transiently induced in G1 by mitogenic factors through the Ras/Raf/ERKs cascade, which is associated with increased CDK4 activity [82,96-100]. Such p21 inductions indeed appear to play a positive role in cell proliferation, as shown by silencing of p21 expression by antisense oligonucleotides or RNA interference [98,101-103]. However, whether the positive role of p21 is mediated by enhanced assembly of D-type cyclin-CDK4 remains to be ascertained. In MCF-7 cells, p21 antisense markedly reduced cyclin D1 expression [102]. Moreover, inhibition by rapamycin of p21 expression, p21 binding to cyclin D1-CDK4 and activation of this complex were not associated with reduction of cyclin D1-CDK4 assembly [104].

The TSH (cAMP)-dependent assembly of active cyclin D3-CDK4 complexes in dog thyrocytes is clearly independent of p21, which is weakly expressed and repressed by TSH [100]. Instead, TSH stimulates the accumulation of p27 [74], which to our knowledge provides a unique such an example in a mitogenic stimulation. Since p27 does associate with cyclin D3-CDK4 without impairing its pRb-kinase activity in TSH-stimulated cells [91], it would constitute a likely candidate as the assembly factor. However, the 2–3-fold increase of p27 levels could not plausibly explain the 10–20-fold increase of cyclin D3-CDK4 complexes induced by TSH. Moreover, inhibition by TGFβ of TSH-stimulated cyclin D3-CDK4 activity is associated with a reduction of the binding of cyclin D3 and CDK4 to p27, but not of the assembly of the cyclin D3-CDK4 complex [85,91]. At variance with Labaer et al [90], we were unable to detect any in vitro assembly activity of p27 for cyclin D3 and CDK4, even in conditions that allow the binding of p27 to pre-assembled cyclin D3-CDK4 complexes ([82], unpublished results). This is in agreement with previous studies showing that abundant p27 in G_0 _cells is unable to assemble ectopically expressed cyclin D3 into CDK4 complexes in serum-starved fibroblasts [78].

Finally, whether the formation of D-type cyclin-CDK complexes would indeed require an assembly factor remains doubtful. The efficient assembly of strongly overexpressed ectopic cyclin D3 and CDK4 in CHO cells cannot be explained by low endogenous levels of p21 or p27 [82]. Similarly, the efficient assembly of D-type cyclins and CDK4 co-produced in insect cell by baculoviral infection is known for a long time [77,82]. We have been unable to detect insect cell proteins stoichiometrically associated with these very abundant complexes (Bockstaele, unpublished results), in agreement with the apparent size of 70 kDa reported for cyclin D2-CDK4 complexes produced in insect cells [89]. However, the native active cyclin D1-CDK4 holoenzyme has been characterized as a complex with a molecular mass ranging from 150 to 200 kDa [105,106].

Other mechanisms should thus be envisaged to explain the regulated formation of D-type cyclin-CDK complexes. Phosphorylations of cyclin D3 do not appear to be involved in TSH-dependent cyclin D3-CDK4 assembly in thyrocytes, since all the main phosphorylated and unphosphorylated forms of cyclin D3 separated by two-dimensional (2D) gel electrophoresis were found in unaltered relative proportions in CDK4 complexes [91]. Moreover, combined mutations of a series of putative phosphorylation sites of cyclin D3 (T283A, T117A, S133A, S264A) did not affect its binding to CDK4 and CDK4 activation in CHO cells (Bockstaele, unpublished data).

#### INK4

Presence of INK4 proteins obviously affects D-type cyclin-CDK association [105]. Parry and collaborators reported that overexpression of p21 can promote the association of D-type cyclins with CDKs by counteracting their binding to INK4 proteins [106]. Conversely, increased expression of p15 in response to TGFβ [107-109], ERK-dependent p16 up-regulation [110], or p18 up-regulation by progestin [111] destabilize D-type cyclin-CDK complexes and mobilize p27. Defective p16 expression in tumor cells is thus very likely to contribute to constitutive cyclin D3-CDK4/6 assembly as we observe it in glioma T98G cells [82]. However, INK4 proteins are not generally down-regulated by mitogenic stimulations, and thus are unlikely involved in stimulated assembly of D-type cyclin-CDK complexes. Interestingly, a phosphorylation (Ser152) of p16 correlates with CDK4 association in human fibroblasts [112]. Regulation of this phosphorylation and its impact on p16-binding to CDK4 remain to be determined.

#### Chaperone proteins

Assembly of D-type cyclin and CDK4 likely depends on proper folding of both subunits. Binding to the chaperone HSP90 via p50^*cdc37 *^stabilizes CDK4 [113,114], competes with p16 and thus would facilitate the assembly of a complex between CDK4 and cyclin D1 [115]. Accordingly, mutations of CDK4 within its ATP-binding domain (G15A and G18A), which preclude Cdc37-CDK4 complex formation, also greatly reduce binding of CDK4 to cyclin D1 [116]. Upon release from Cdc37-HSP90 interaction, properly folded newly synthesized CDK4 could thus directly assemble with D-type cyclins, preferentially to the more inert pool of INK4-bound CDK4. Once again, mitogenic regulation at this level has not been defined. In another recent development, Diehl and collaborators have identified Hsc70 as at least one of the probable missing component of the active cyclin D1-CDK4 holoenzyme of NIH 3T3 cells [117]. Hsc70-cyclin D1 interaction is stimulated by serum and would promote stabilization of newly synthesized cyclin D1, thereby increasing its availability for assembly with CDK4. Nevertheless, Hsc70 does not appear to assemble the cyclin D1-CDK4 complex. Furthermore, interaction between Hsc70 and endogenously expressed cyclin D1 was hardly detectable [117]. In dog thyrocytes stimulated by TSH, we also easily identified Hsc70 from two-dimensional gel separation of co-immunoprecipitations by cyclin D3 and CDK4 antibodies. Unfortunately, unspecific association of Hsc70 to mock immunoprecipitations (done with normal mouse serum) that could not be displaced by high salt washings, as well as our failure to detect endogenously expressed cyclin D3 and CDK4 in Hsc70 immunoprecipitations, have prevented us from further investigating this interaction (Paternot, unpublished results).

#### SEI-1

p34^*SEI-1*^, also known as TRIP-Br1, has been separately identified as a p16 binding protein [118] and as a transcriptional regulator that can functionally contact DP-1 and co-activate E2F-1/DP-1 transcriptional activity [119]. NIH 3T3 cells engineered to overexpress SEI-1 are anchorage-independent and tumorigenic, and amplification of *SEI-1 *gene is commonly detected in ovarian cancers [120]. SEI-1 is induced by serum, and its ectopic expression allows the formation of active CDK4 complexes that contain both p16 and cyclin D1. SEI-1 might thus facilitate the assembly of cyclin D1-CDK4 complexes by counteracting inhibitory effects of INK4 proteins [118]. Physiological relevance of this interesting mechanism remained doubtful, however, since complexes containing both p16 and cyclin D1 are not generally observed [60]. Indeed, the same authors showed in a subsequent study that expression of p16 blocks the pRb-kinase activity associated with ectopically expressed SEI-1 [94]. Ablation of SEI-1/TRIP-BrI and TRIP-Br2 expression suppresses serum-induced cyclin E expression and prevents S-phase entry [121]. Whether this results from an impairment of CDK4 complex activation or of E2F-dependent transcription has yet to be defined.

#### Gankyrin

The oncoprotein gankyrin [122] is a seven-ankyrin-repeat protein that interacts with pRb, increases phosphorylation of pRb by CDK4, and also increases the binding of p53 to MDM2 [123]. In hepatocytes, gankyrin expression is increased by mitogenic stimulation [124]. A search for interaction partners of transfected gankyrin has identified CDK4 [125], and gankyrin competes with p16 for binding to CDK4 in reconstitution experiments [126]. It is not known if gankyrin binds to monomeric CDK4 or CDK4 complexed with D-type cyclins.

#### Fbxo7

Recently, the F-box protein Fbxo7 was identified in a two-hybrid yeast screen using the viral cyclin of *Herpesvirus saimiri *as a bait [127]. Fbxo7 turned out to specifically interact in vitro and in intact cells with CDK6 (but not CDK4). Whereas knockdown of Fbxo7 reduces p27 expression and association of CDK6 with D-type cyclins, Fbxo7 fails to assemble cyclin-CDK6 complexes in vitro, and ectopic expression of Fbxo7 only moderately enhances CDK6 association with D-type cyclins and pRb-kinase activity. Despite this rather modest effect, overexpression of Fbxo7 transforms NIH 3T3 cells in a CDK6-dependent manner [127], which does not rule out an implication of other Fbxo7 interactors. Regulation of Fbxo7 by mitogenic factors was not reported.

To conclude, the assembly of D-type cyclin-CDK4/6 complexes is a highly regulated process. Only p21 or p27 have been found to be stoichiometrically present in these complexes. However, they are not required for the assembly of D-type cyclin-CDK complexes, though they can obviously stabilize them. INK4 proteins clearly restrict cyclin-CDK assembly, but regulation at this level has not been determined. Mainly in overexpression systems, other interesting interactors of CDK4/6 or D-type cyclins have been demonstrated. They could influence the conformation of the cyclin or CDK subunit, and facilitate either the release of CDK4/6 from its complex with inhibitory INK4 proteins or the association of the CDK with the cyclin. Nevertheless, much additional experimental evidence is needed to demonstrate the physiological relevance and regulation of these new interactions. Molecular masses of the D-type-cyclin-CDK4/6 and their complex with p21/p27 are predicted to be about 70 and 100 kDa, respectively. Such complexes do exist in intact cells. On the other hand, the missing component(s) of the abundant 150–200 kDa active CDK4/6 complexes that have been demonstrated in various cell systems remain to be identified.

### Regulated nuclear translocation of D-type cyclin-CDK4 complexes

D-type cyclin-CDK4/6 complexes should accumulate in the nucleus in order to phosphorylate their nuclear substrates including pRb, p107 and p130. Moreover, the activity of CDK4/6 requires their activating phosphorylation on Thr172/177 by the CDK-activating kinase (CAK), which is a nuclear holoenzyme (but see discussion below). Accordingly, a mutant of cyclin D1 that assembles with CDK4 but prevents its nuclear localization (but also its activation by CAK in vitro) dominantly inhibits the ability of NIH 3T3 cells to enter S phase [128]. Conversely, ectopic expression (together with CDK4) of a nucleus-targeted variant of cyclin D1 but not of wild-type cyclin D1 promotes reentry of cardiomyocytes into cell cycle, suggesting a critical role of cyclin D1 nuclear import [129]. Recently, the oncogenic potential of mutations that prevent the nuclear export of cyclin D1 has been demonstrated [54,130]. Nuclear translocation of cyclin D1 and CDK4 during mitogenic processes has been observed in vivo, including in rat liver regeneration [131] and in the estrogen-induced proliferation of uterine epithelium [132].

D-type cyclins and CDK4/6 do not possess obvious nuclear localization sequences. Furthermore, depending on its Thr286 phosphorylation by GSK3β which allows its binding to the exportin CRM1, cyclin D1 is exported to the cytoplasm during S-phase in fibroblasts [133]. Other CDK4/6 binding proteins including INK4 proteins [34,107] and Cdc37 [113] are mostly cytoplasmic proteins. By contrasts, Cip/Kip proteins contain a well characterized bipartite nuclear localization signal (NLS) in their C-terminus. Enforced expressions of p21, p27 and p57 were thus demonstrated to localize D-type cyclin-CDK complexes to the nucleus [82,90,107,134]. These proteins really determine the location of CDK4, as the deletion of the NLS of p21 and p27 relocalize CDK4 to the cytoplasm [82,90,107]. In NIH 3T3 cells, p21 promotes the nuclear accumulation of cyclin D1 by preventing its association with CRM1 [135]. Like its assembly [79], the nuclear accumulation of cyclin D1-CDK4 appears to depend on MEK activity in NIH 3T3 cells [136].

To our knowledge, dog thyrocyte primary cultures have provided the first example of a nuclear translocation of endogenously expressed CDK4 in response to various mitogenic stimulations, including TSH and cAMP, growth factors and phorbol esters [70]. CDK4 nuclear translocation elicited by TSH (cAMP) correlates in individual cells with G1 and S-phase progression [70] and depends on the presence of comitogenic factors (insulin and carbachol) [83,84]. In thyrocytes stimulated by growth factors, CDK4 nuclear import perfectly correlates in individual cells with binding to cyclin D1 and up regulated nuclear p21 [45], whereas it is associated with cyclin D3 and nuclear accumulation of p27 in TSH-stimulated cells [85,91]. The inhibition by TGFβ of TSH-elicited nuclear import of both CDK4 and cyclin D3 is explained by their reduced association with nuclear p27. Interestingly, the TSH-dependent assembly of cyclin D3-CDK4 complexes is not affected by TGFβ, which dissociates the nuclear import from the assembly, and points out the critical role of p27 in the subcellular location but not in the assembly of cyclin D3-CDK4 [85,91]. Intriguingly, the nuclear translocation of cyclin D3 in thyrocytes is associated with the unmasking of its DCS-22 epitope (aa 241–260), which suggests a conformational change of cyclin D3 or a modification of its interaction with other proteins [70,85]. At variance with the situation described for the export of Thr286-phosphorylated cyclin D1 in fibroblasts [133], cyclin D3 steadily accumulates in nuclei during S and G_2_-phases [70], and the phosphorylation of cyclin D3 at Thr283 by GSK3β does not signal its nuclear export [137].

In many cell systems, late G_1 _phase progression depends on the activity of the calcium-binding protein calmodulin. Pharmacological inhibition of calmodulin in NRK fibroblasts inhibits the activity of cyclin D1-CDK4 complexes by inducing their translocation to the cytoplasm, providing another early example of regulation of D-type cyclin-CDK4 complex localization dissociated from the modulation of its assembly [138]. This calmodulin-dependent nuclear accumulation of cyclin D1-CDK4-p21 complexes appears to depend on a direct calcium-dependent binding of p21 to calmodulin [139].

As p21 and p27 clearly determine the subcellular location of D-type-cyclin-CDK4 complexes, posttranslational modifications that alter the nuclear location of p21 or p27 should also affect the localization of CDK4 and its activity. The impact on p21 subcellular location of its Thr145-phosphorylation by overactivated Akt or Pim1 remains controversial [140-143]. The major phosphorylation of p27^kip1 ^at Ser10 [144] has been reported to cause the nuclear export of p27^kip1 ^during G1 phase progression in fibroblast cell lines [145-147]. The inactivation of p27^kip1 ^by its cytoplasmic mislocalization in different breast cancer cell lines is caused by p27^kip1 ^phosphorylation within the NLS at Thr157 by overactivated Akt/PKB [148-150]. Thr157 phosphorylation of p27 by cytoplasmic Akt, which prevents p27 binding to importin α and thus nucleus re-entry, appears itself to depend on prior Ser10-phosphorylation of p27 required for its nuclear export [151]. This relocalization of p27 into the cytoplasm is believed to critically contribute to the activation of cyclin E-CDK2. Unfortunately, its impact on CDK4 complex localization and activity was not investigated in the above referenced studies. Ser10 phosphorylation of p27 is unlikely to be sufficient for nucleus export. Nuclear p27 is abundantly phosphorylated at Ser10 in dog thyrocytes and p27-transfected CHO cells [82,91]. In dog thyrocytes, the phosphorylation profile of p27 (as resolved by isoelectric focusing separation) and its Ser10-phosphorylation are not consistently modulated by cAMP, TGFβ, insulin, growth factors, or pharmacological inhibition of MEK- or PI3-kinase-dependent signalling, and all the (un)phosphorylated forms of p27 are found in unaltered proportion in cyclin D3-CDK4 complexes [82,91].

To conclude, the instrumental role of the up-regulation by mitogenic treatments of either p21 or p27 in the required nuclear import of D-type cyclin-CDK4 complexes is well demonstrated in a variety of cell systems. It appears to be dominant over mechanisms that promote nucleus export of CDK4 and D-type cyclins, including the GSK3β-dependent phosphorylation of cyclin D1. A significant role for posttranslational modifications of p21 and p27 in the regulation of this process has yet to be firmly demonstrated.

### Regulated activity of D-type cyclin-CDK4 complexes

The assembly of D-type cyclin-CDK4/6 complexes and even their nuclear location are not sufficient for their catalytic activity. Upon mitogenic stimulation of quiescent cells, appearance of the pRb-kinase activity of CDK4 is often delayed compared to the formation of D-type cyclin-CDK4 complexes [80,82,131,152,153]. Both processes are clearly dissociated in other situations, which include the inhibition of the activity of D-type cyclin-CDK4 complexes by cAMP or rapamycin in mouse macrophages [75], contact inhibition in 3Y1 rat fibroblasts [154], senescence of human fibroblasts [155], antiestrogen treatment of MCF-7 breast cancer cells [156], calmodulin inhibitors in human fibroblasts [157], and arrest of cAMP-dependent mitogenic stimulation in dog thyrocytes by forskolin deprivation [158,159], TGFβ [91] or inactivation of Rho proteins by *Clostridium *toxin B [160].

Inhibition by p21 or p27 is the most generally proposed mechanism to explain the inactivity of D-type cyclin-CDK4 complexes [59,63,161]. Thus, the activation of cyclin D1-CDK4 complexes during G1 phase progression depends on p27 disappearance [80,153], and is prevented by up-regulation of p27 [75,154] or p21 [155,156]. This obviously contrasts with the positive roles (stabilization and nuclear anchoring) of these "inhibitors" on D-type cyclin-CDK4 complexes, and the frequent observation that they can support the pRb-kinase activity of CDK4 [162]. Whereas G_1 _arrest by cAMP in mouse macrophages is associated with p27 up-regulation which inhibits cyclin D1-CDK4 activity [75], in the cAMP-dependent G_1_-phase progression of dog thyrocytes, an apparently similar elevation of p27 concentration allows the nuclear import and activation of cyclin D3-CDK4 complexes, and both processes are prevented by TGFβ which inhibits the binding of this complex to p27 [91]. Similarly, inhibition of cyclin D1-CDK4 activity has been explained either by increased [156] or decreased [104] binding of p21.

Labaer et al [90](in the case of p21 but not of p27) and Blain et al [89](in the case of p27 but not of p21) have interestingly shown that such opposite roles of CDK "inhibitors" could depend on their stoichiometry relative to a D-type cyclin in CDK4 complexes, as initially shown for the dual action of p21 on cyclin A-CDK2 [163]. Nevertheless, structural studies indicate that cyclin A-CDK2 complexes can accommodate only one molecule of p21 or p27, which fully inhibits the activity [164-166]. Moreover, according to Pledger and collaborators, only the minor fraction of cyclin D3-CDK4 complexes devoid of Cip/Kip proteins is active [93,95]. Our analysis of the impact of graded concentrations of p27 relative to cyclin D3 and CDK4 in CHO and Sf9 cells and in vitro, as well as the comparison of their relative expression levels in native systems where p27 supports or prevents CDK4 activity, fully confirm the "stoichiometric" model [82]. This implies two types of cyclin D3-CDK4-p27 complexes depending on relative p27 concentrations: the first comprising a low stoichiometry binding of p27 and displaying a pRb-kinase activity, and the second, inactive due to additional p27 molecule(s). Whereas the association of p27 to CDK complexes is generally believed to depend on its binding to the cyclin [60,166], we have found both in Sf9 and CHO cells, that p27 can avidly form binary complexes with CDK4 in the absence of a D-type cyclin [82]. In native systems, we also observed a persistence of p27-CDK4 complexes after the disappearance of labile D-type cyclins provoked by protein synthesis inhibition [82,159]. This association of p27 with cyclin-free CDK4 in intact cells is consistent with the model of different p27-binding modes permitting the association of several p27 molecules to D-type cyclin-CDK4 complexes.

The D-type cyclins/p27 ratio can thus contribute to determine the cell responsiveness to mitogens or growth inhibitory factors, which in turn act to modify this equilibrium to levels that allow, or prevent, the concerted activation of CDKs leading to S-phase entry [162]. In some circumstances, as in quiescent dog thyrocytes which express high amounts of cyclin D3 [70] but low levels of p27 and p21, p27 (in response to TSH) or p21 (in response to growth factors [45]) rather than D-type cyclins may have to be up-regulated to facilitate CDK4 activation. Similarly in mammary gland and prostate of p27-null mice, epithelial cell proliferation was reported to be impaired, whereas p27 haplo-insufficiency accelerated cyclin D1-dependent transformation, which was prevented by normal p27 expression [167-169].

Some modulations of the activity of D-type cyclin-CDK4 complexes cannot be explained by modifications of their association with p21 or p27 [82,157,158,160]. For instance, in dog thyrocytes the withdrawal of the cAMP stimulation rapidly arrests the entry of cells in S-phase and pRb phosphorylation but does not reverse the formation of nuclear cyclin D3-CDK4-p27 complexes [84,158].

### Regulated phosphorylation of CDK4

Phosphorylation is the least studied level of regulation of CDK4. An inhibitory phosphorylation of CDK4 on Tyr17 was reported in UV irradiation-induced G_1 _arrest [170] or during cell's arrest in quiescence [171] or in response to TGFβ [172]. However, the group of Sherr failed to detect a tyrosine-phosphorylation of CDK4 [77,173], in agreement with the observation that CDK4, unlike CDK1 and CDK2, is not an in vitro substrate of the Wee1 CDK tyrosine kinase [174]. On the other hand, by analysing human D-type cyclin-CDK4 expressed in insect cells through baculoviral infection, Kato et al. have demonstrated that the activity of CDK4 requires its phosphorylation on Thr172 [77] within the activation loop. Thr172-phosphorylation of CDK4 requires its binding to a D-type cyclin, while T172A mutation of CDK4 does not affect its binding to the cyclin [77]. Furthermore, this group showed that mammalian cell extracts possess a CDK4-activating kinase activity which was ascribed to CAK (cyclin H-CDK7) on the basis of the immunodepletion of this in vitro activity by a polyclonal CDK7 antibody [175]. Nevertheless, the activity of nuclear CAK (CDK7) complexes has generally been found to be constitutive and non-regulated during cell cycle or mitogenic stimulations [82,175-177]. Thr172-phosphorylation of CDK4 is thus assumed to passively result from CDK4 binding to a cyclin and subsequent nuclear import. When it is inhibited within the cyclin D1-CDK4 complex by cAMP or contact inhibition, this was ascribed to increased binding of p27 [75,154], which has been found to prevent the activating phosphorylation of CDKs by CAK in vitro [75,178-181].

Despite the fact that it is required for CDK4 activity [77,82], the activating Thr172 phosphorylation of CDK4 has been infrequently investigated because of lack of methodological tools. We have recently shown that the high-resolution power of the two-dimensional (2D) gel electrophoresis (isoelectric focusing and SDS-PAGE) allows one to separate several phosphorylated and non-phosphorylated forms of CDKs and to visualize their relative proportions in the different complexes [82,91,182]. At variance with CDK2 [182], only one major single phosphorylated form of CDK4 is observed in a variety of cell types, including dog and human thyrocytes, human fibroblasts, T98G glioma cells, Rat2 fibroblasts,... ([82,91,100], and our unpublished results). We have identified this main phosphorylated form of CDK4 as comprising the activating Thr172 phosphorylation, by the analysis of the CDK4 T172A mutant, in vitro phosphorylation by recombinant CAK, as well as the utilization of first samples of a trial production (not yet commercialized) of a Thr172 phosphospecific CDK4 antibody by Cell Signalling Technology Inc. [82]. These tools were also useful for the analysis of the corresponding (Thr177) phosphorylation of CDK6 [82]. In those cells, no stoichiometrically (biologically) significant phosphorylated form of CDK4 (except a very minor form that comprises the Thr172 phosphorylation and a possible second undefined one) could contain the inhibitory Tyr17 phosphorylation. In human fibroblasts (even after UV irradiation (Kooken, unpublished results)), the lack of Tyr17-phosphorylated CDK4 contrasts with the major phosphorylation of CDK2 on Tyr15 [182], implying that activation of CDK4 and CDK2 must very differently depend on Cdc25 phosphatases.

As demonstrated by 2D-gel separation of CDK4, the cAMP-dependent cell cycle regulation and its inhibition by TGFβ in dog thyrocytes have provided the first examples of a regulation of the activating Thr172-phosphorylation of CDK4 independently of changes in its association with cyclins and CDK « inhibitors » [82,91,159]. Upon arrest of forskolin stimulation, which induces the rapid inactivation of cyclin D3-CDK4-p27 complexes without preventing their continued formation, Thr172-phosphorylation of CDK4 disappears from the cyclin D3 complexes. The association of CDK4 with cyclin D3 and its activating phosphorylation within this complex are thus separately controlled by cAMP [159]. The strong inhibition by TGFβ of the activity of cyclin D3-CDK4 complexes in TSH (cAMP)-stimulated dog thyrocytes is also explained by decreased Thr172-phosphorylation of CDK4, even within the residual cyclin D3-CDK4 complexes that remain associated with p27 [82,91]. The activating phosphorylation of CDK4 thus integrates the opposite cell cycle controls by cAMP and TGFβ after formation of cyclin D3-CDK4 complexes. Furthermore, in T98G cells and Rat2 cells (unpublished data), serum stimulation strongly activates constitutively formed cyclin D3-CDK4 complexes at least in part by directly stimulating the Thr172-phosphorylation of CDK4 [82]. This would generalize the new concept that Thr172-phosphorylation could be a latest regulated step that determines the catalytic activity of CDK4, the phosphorylation of Rb family proteins and thus the passage through the G_1 _phase restriction point.

In all those examples, modifications of p27-binding to CDK4 complexes were unlikely to explain the modulations of CDK4 phosphorylation. Indeed, the proportion of Thr172-phosphorylated CDK4 is similarly enriched in D-type cyclin complexes and in CDK4 bound to p27 or p21 [45,82,91,159], even at high relative expression levels of p27 that preclude CDK4 activity [82]. At variance with previous conclusions [75], p27 does not inhibit the activity of D-type cyclin-CDK4 complexes by preventing the activating phosphorylation of CDK4.

### Regulated CDK4-activating kinase(s) ?

The mechanisms involved in the regulation of the phosphorylation of cyclin D3-bound CDK4 remain enigmatic. In mammalian cells, the major CAK activity that phosphorylates CDK4/6, CDK2 and CDK1 is considered to be constituted of cyclin H-CDK7-MAT1, which are also subunits of transcription factor II H [181,183,184]. In principle, (anti)mitogenic signalling cascades might regulate the activity of CAK, its substrate specificity or its access to CDK4. However, we got no evidence of a regulation of CAK (CDK7 complex) expression or activity in dog thyrocytes and T98G cells [82], as most often reported [175-177]. This might suggest that constitutive CAK (CDK7) complexes could not be instrumental in the regulated phosphorylation of CDK4. In addition to the observation discussed above that Thr172-phosphorylation of CDK4 is not prevented by inhibitory concentrations of p27, other arguments call for a reconsideration of CDK4-activating kinase(s). Whereas CDK7 activity is nuclear, deletion of p27 NLS sequence relocalizes most of cyclin D3-CDK4-p27 complexes in the cytoplasm without impairing phosphorylation of p27-bound CDK4 [82]. As analysed in the same cyclin D3 co-immunoprecipitations in T98G glioma cells, serum stimulates the activating phosphorylation of CDK4 but not that of the related CDK6 [82]. Nevertheless, the latter appears as a better substrate for recombinant CAK (cyclin H-CDK7-Mat1) in the same experiments [82], consistent with observations by others who succeeded to readily phosphorylate CDK2 and CDK6, but not different CDK4 preparations, using recombinant CAK [153,180].

Although CAK (CDK7) *can *unambiguously phosphorylate and activate cyclin D3-bound CDK4 in vitro, other regulated CDK4-activating kinase(s) might thus remain to be discovered. They might even be different in distinct mitogenic stimulations and selectively target CDK4 complexed to cyclin D3 or cyclin D1. Some observations cannot be easily explained by only one kinase activating the different D-type cyclin-CDK4 complexes. Indeed, how could TGFβ selectively inhibit the activating phosphorylation of CDK4 bound to cyclin D3 in dog thyrocytes stimulated by TSH, without affecting the activity and phosphorylation of CDK4 stimulated by EGF [91]? How could EGF stimulate the phosphorylation and activity of CDK4 bound to cyclin D1 in human thyrocytes, but not the activity of abundant cyclin D3-CDK4 complexes, which were specifically activated by TSH [100]?

Recent studies have also pointed out the Thr160 phosphorylation of CDK2 as a direct target of treatments that prevent S-phase entry [185-188]. In some of these studies, the activation of CDK4 [185,186] and/or the in vitro assayed activity of CDK7 [185,188] remain unaffected, leading their authors to suggest the involvement of distinct CAK activities. In *D. melanogaster*, CDK7 is required for the activation of CDK1 complexes but not for phosphorylation and activation of CDK2-cyclin E [189]. At variance with nuclear cyclin H-CDK7 of animal cells, the cytoplasmic monomeric Cak1p of budding yeast [190] preferentially phosphorylates monomeric CDK2 and CDK6 in vitro, and CDK inhibitors including p27 do not block this activity [180]. Several monomeric CAKs with distinct substrate specificities coexist with CDK7 orthologs in plants [191,192]. In human cells, a small distinct CAK activity was enriched [193], and a candidate nuclear p42 CAK was recently cloned [194]. Although the CDK2 phosphorylating activity of this p42 "CAK" has been disputed recently [195], it seems that the debate about the nature of mammalian CAK(s) [181,183,184] is not coming closer to its end.

## Conclusion

**Figure 1:**
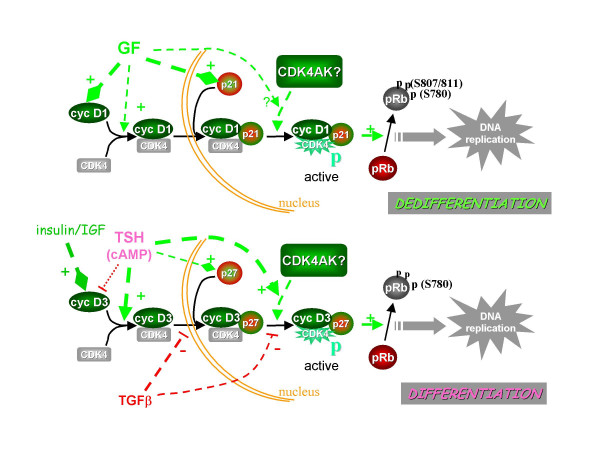
**CDK4 regulation: the example of canine thyroid primary cultures**. Two distinct mitogenic modes coexist in dog thyrocytes and are differentially associated with differentiation expression. Growth factors (GF) activate CDK4 as in other systems, mainly by inducing cyclin D1 and also p21, which stabilizes the cyclin D1-CDK4 complex in the nucleus. The differentiation-associated cell cycle activation by TSH and cAMP is adjunctive to this basic control. It utilizes cyclin D3 synthesized in response to insulin or IGF-I, and p27. cAMP activates CDK4 by promoting the assembly of the cyclin D3-CDK4 complex, its association with nuclear p27, and finally the activating Thr172-phosphorylation of CDK4 within this complex by an undefined CDK4 activating kinase (CDK4AK). Activation of CDK4 complexed to cyclin D1 or cyclin D3 in these parallel mitogenic stimulations leads to partially different site-specificity of pRb-kinase activity. In this system, TGFβ selectively inhibits the cAMP-dependent activation of cyclin D3-CDK4, not by impairing the formation of this complex, but by preventing its binding to nuclear p27, as well as by inhibiting CDK4 phosphorylation within residual p27-bound cyclin D3-CDK4 complexes. The dog thyrocyte model illustrates the dissociation of the regulated assembly of D-type cyclin-CDK4 complexes from the accumulation of their subunits, the dissociation of the nuclear import of these complexes from their assembly, and the dissociation of the rate-limiting phosphorylation of CDK4 from the formation and nuclear accumulation of the D-type cyclin-CDK4-p27/p21 holoenzyme. Diamond arrowheads represent inductions; the other dashed arrows are activations (+) or inhibitions (-).

The activation of CDK4 is a peculiarly complex process requiring different successive steps, which are distinguished by their independent regulations. This allows to better understand how CDK4 can function at the restriction point as a master integrator of various mitogenic and antimitogenic controls of the cell cycle. A few models, including the physiologically relevant example of the thyroid primary culture system, have allowed to dissociate the regulated assembly of D-type cyclin-CDK4 complexes from the accumulation of their individual partners, the nuclear import of these complexes from their assembly, and the activating phosphorylation of CDK4 from the formation and nuclear accumulation of the D-type cyclin-CDK4-p27/p21 holoenzyme (Figure 1). We have identified this last step as an ultimate regulatory target determining CDK4 activity, pRb phosphorylation and the passage through the G_1 _phase restriction point. Some evidence is emerging that the activation process of the six possible cyclin D1/2/3-CDK4/6 complexes could be subject to partly different regulatory mechanisms. Cyclin D3-CDK6 complexes appears to be especially resistant to inhibition by p16 [196] and p27 [197]. In T98G glioma cells, serum stimulates the phosphorylation of cyclin D3-bound CDK4 but not cyclin D3-bound CDK6 [82]. In thyrocytes, the distinct mitogenic pathways of growth factors or TSH appear to be channeled to preferentially activate and utilize CDK4 complexed to either cyclin D1 or cyclin D3 [45,70,100] (Figure 1). This leads to partly different phosphorylations of pRb, which might differentially impact pRb function in the transcriptional regulation of genes involved in cell cycle or differentiation [45,100].

The visualization of the phosphorylation profiles, as resolved using 2D-gel electrophoresis, of CDK4 [82], CDK2 [182] and CDK1 (Kooken and Coulonval, unpublished results) has illustrated the different logics of their regulations. Cyclin-CDK1/CDK2 accumulate as a reservoir of inactive complexes containing both activating (Thr161/160) and inhibitory (Tyr15 and Thr14) phosphorylations until their activation by dephosphorylation of the inhibitory residues by Cdc25 phosphatases. On the other hand, CDK4 activity could not be generally restricted by substantial Tyr-phosphorylation, but by lack of activating phosphorylation, at variance with the corresponding phosphorylations of CDK2/1 by CAK which are not thought to be rate-limiting.

CDK4 activity is crucial in various tumorigenesis models and appears as a very attractive target for cancer therapy [198]. As discussed in this overview, increased expression of a D-type cyclin is clearly not sufficient to activate CDK4, which likely explains the lack of relationship between cyclin D1 overexpression and CDK4 activity in neoplastic epithelial cell lines [199]. Mechanisms responsible for the regulation of the assembly and activation of D-type cyclin-CDK complexes are crucial but remain largely enigmatic. They constitute obvious targets for oncogenic mechanisms. Their elucidation thus remains a major challenge that might lead to the discovery of new therapeutic targets.

## Competing interests

The author(s) declare that they have no competing interests.
